# The value of routine measurement of serum calcitonin on insufficient, indeterminate, and suspicious thyroid nodule cytology

**DOI:** 10.17305/bjbms.2021.5756

**Published:** 2021-05-21

**Authors:** Muhammed Erkam Sencar, Sema Hepsen, Murat Calapkulu, Hayri Bostan, Davut Sakiz, Ilknur Ozturk Unsal, Hakan Duger, Muhammed Kizilgul, Bekir Ucan, Tugba Taskin Turkmenoglu, Mustafa Ozbek, Erman Cakal

**Affiliations:** 1 Department of Endocrinology and Metabolism, University of Health Sciences, Diskapi Yildirim Beyazit Training and Research Hospital, Ankara, Turkey; 2 Department of Pathology, University of Health Sciences, Diskapi Yildirim Beyazit Training and Research Hospital, Ankara, Turkey

**Keywords:** Nodular thyroid disease, calcitonin, medullary thyroid cancer, cytology

## Abstract

Routine calcitonin measurement in patients with nodular thyroid disease is rather controversial. The aim of this study was to evaluate the contribution of serum calcitonin measurement in the diagnostic evaluation of thyroid nodules with insufficient, indeterminate, or suspicious cytology. Out of 1668 patients who underwent thyroidectomy with the diagnosis of nodular thyroid disease and were screened, 873 patients with insufficient, indeterminate, or suspicious fine-needle aspiration biopsy results were included in the study. From the total number of patients in this study, 10 (1.1%) were diagnosed as medullary thyroid cancer (MTC) using histopathology. The calcitonin level was detected to be above the assay-specific cutoff in 23 (2.6%) patients ranging between 6.5 and 4450 pg/mL. While hypercalcitoninemia was detected in all 10 MTC patients, a false positive elevation of serum calcitonin was detected in 13 patients (1.5%). Of the MTC group, seven patients had cytology results that were suspicious for malignancy (Bethesda V), one patient’s cytology showed atypia of undetermined significance (Bethesda III), and two patient’s cytology results were suspicious for follicular neoplasm (Bethesda IV). Among the cases with non-diagnostic cytology (Bethesda I), none of the patients were diagnosed with MTC. In conclusion, routine serum calcitonin measurement can be performed in selected cases rather than in all nodular thyroid patients. While it is reasonable to perform routine calcitonin measurement in patients with Bethesda IV and Bethesda V, this measurement was not useful in Bethesda I patients. In Bethesda III patients, patient-based decisions can be made according to their calcitonin measurement.

## INTRODUCTION

Medullary thyroid cancer (MTC) originates from thyroid C cells and accounts for 1–2% of thyroid cancers [1]. It is a rare type of cancer, and the prognosis is usually poor because most patients are diagnosed at an advanced stage or after thyroidectomy [2]. Patients with MTC usually have metastases at the time of diagnosis, and it is too late to perform curative surgery [3]. Early diagnosis and successful radical surgery is very important in terms of disease prognosis [4]. The production of calcitonin, a 32 amino acid long polypeptide hormone, is a characteristic feature of this tumor. Therefore, calcitonin measurement plays an important role in both diagnosis and follow-up of MTC patients [5,6] Many studies have shown that routine measurement of serum calcitonin may lead to earlier detection of MTC in patients with thyroid nodules [4,7-12]. The American Thyroid Association (ATA) has remained neutral on the subject of routine calcitonin measurement in thyroid nodule follow-up, leaving the decision up to the clinicians [13]. The aim of this study was to evaluate the contribution of routine serum calcitonin measurement to the initial diagnostic evaluation of thyroid nodules with insufficient, indeterminate, or suspected cytology in fine-needle aspiration biopsy (FNAB) (The Bethesda System categories: I, III, IV, V) [14].

## MATERIALS AND METHODS

In this retrospective cohort study, clinical data were obtained from the prospectively maintained thyroidectomy (total/subtotal) patient database held within the Department of Endocrinology and Metabolism. A total of 1668 patients who underwent thyroidectomy with the diagnosis of nodular thyroid disease between 2014 and 2019 were screened. Of these patients, 569 patients with benign or malignant FNAB results (Bethesda category II and VI) and 226 patients with missing calcitonin results before thyroidectomy were excluded from the study. Thus, the study included 873 patients with insufficient, indeterminate, and suspicious FNAB results. Serum calcitonin was measured in all the patients included in the study and all patients underwent FNAB before thyroidectomy. In patients with Multinodular Goiter (MNG), FNAB was applied to the largest nodule or to the highest-risk nodule. Fine-needle aspiration (FNA) smears were air dried and stained with May Grunwald Giemsa. Cytological diagnosis was made according to the Bethesda system [14] by an experienced cytopathologist (TTT). The patients included in the study were those with the following FNAB results; non-diagnostic (ND)/unsatisfactory (I); atypia of undetermined significance/follicular lesion of undetermined significance (AUS/FLUS) (III); follicular neoplasm/suspicious for follicular neoplasm (FN/SFN), Hurthle cell neoplasm/suspicious for Hurthle cell neoplasm (IV); and suspicious for malignancy (V).

The decision to perform surgery was made for patients with nodular thyroid disease, patients with oversized nodules, patients with obstructive symptoms, and patients with anxiety due to their nodules. Thyroidectomy specimens were fixed with 10% buffered formaldehyde. After routine tissue processing, tissues were embedded in paraffin and stained with hematoxylin-eosin. Calcitonin immunohistochemical staining was performed by an automatized stainer (Ventana BenchMark XT, Roche Diagnostics, Switzerland) to confirm the medullary cancer diagnosis, whenever it was needed. Calcitonin was measured with the chemiluminescence immunoassay (Immulate 2000 Siemens) with analytical sensitivity of 1 pg/mL. For calcitonin, a level of <5 pg/mL was considered normal. The calcitonin stimulation test was not used in our clinic, but the FNAB cytology of thyroid specimens was re-examined in patients with a high calcitonin level and the decision for more invasive surgery was taken if necessary. Thyroid ultrasound (US) was performed using a high-resolution B-mode US device (Hi vision preius; Hitachi, Tokyo, Japan) with a 13-MHz linear array transducer by endocrinologists experienced in the use of ultrasonography.

### Ethical statement

The study protocol was approved by the Ethics Committee of the University of Health Sciences, Diskapi Yildirim Beyazit Training and Research Hospital, Ankara, Turkey (107/04). Informed consent was obtained from all individual participants included in the study. The study was conducted in accordance with the Declaration of Helsinki.

### Statistical analysis

Categorical data were expressed in frequencies and percentages, and continuous variables as mean ± standard deviation and median (range) values according to conformity to normal distribution parameters. In the comparisons of continuous variables, the independent sample *t*-test was applied if data were normally distributed; otherwise, the Mann–Whitney U-test was used. Chi-square analysis was used to evaluate relationships between categorical variables. *p* < 0.05 was accepted as statistically significant.

## RESULTS

**FIGURE 1:**
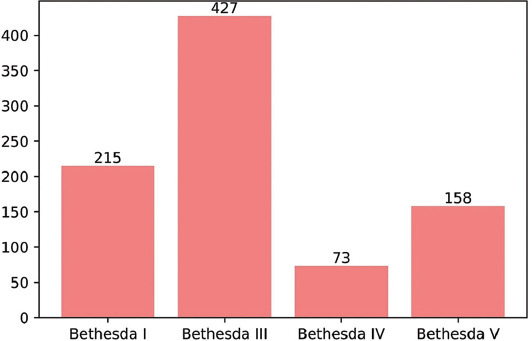
The distribution of cytology results according to the Bethesda system.

**FIGURE 2:**
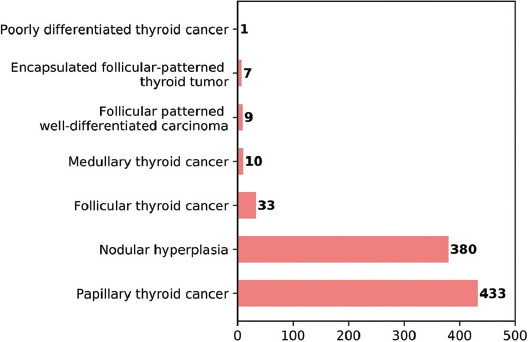
The distribution of pathology results after thyroidectomy.

**FIGURE 3:**
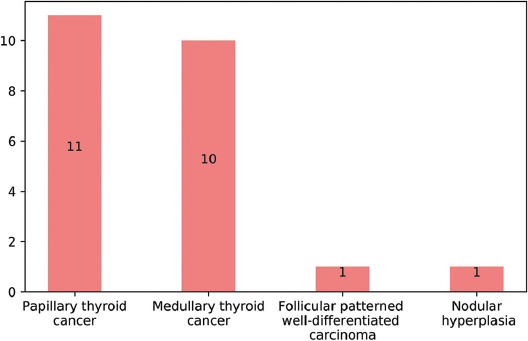
The distribution of the pathology results of patients with a high calcitonin level.

Evaluation was made of a total of 873 patients, comprising 752 (86%) females and 121 (14%) males with a mean age of 48 ± 12. Of these patients, 684 (78.4%) presented with MNG and 189 (21.6%) patients presented with a solitary thyroid nodule before thyroidectomy. The median number of nodules in all patients was 2 (1-15) and the median size of the longest dimension of nodules was 1.8 cm (0.3-8.1). According to the FNAB results, the distribution of patients was as follows; 427 (49%) AUS/FLUS (Bethesda III), 215 (24.7%) ND/unsatisfactory (Bethesda I), 158 (18%) suspicious for malignancy (Bethesda V), and 73 (8.3%) SFN or Hurthle cell neoplasm (Bethesda IV) (Figure 1). Lobectomy was applied to 71 (8%) patients, and the remaining 802 (92%) patients underwent total thyroidectomy. According to the pathology results after thyroidectomy, the distribution of patients was as follows; 380 (43.6%) had nodular hyperplasia, 433 (49.6%) had papillary thyroid cancer (PTC), 33 (3.8%) had follicular thyroid cancer (FTC), 10 (1.1%) had MTC 9 (1%) had follicular patterned well differentiated carcinoma (not otherwise specified, [NOS]), 7 (0.8%) had encapsulated follicular-patterned thyroid tumors, and 1 (0.1%) had poorly differentiated thyroid cancer (Figure 2). The calcitonin level was detected to be above the assay-specific cutoff value in 23 (2.6%) patients ranging between 6.5 and 4450 pg/mL. The clinical and histological data of these 23 patients are given in Table 1. According to the calcitonin results, the decision for more invasive surgery was made for eight of these 23 patients. In addition to total thyroidectomy, central and total lateral lymph node dissection was performed in five patients due to high calcitonin level, while central lymph node dissection was performed in three patients. False positive elevation of serum calcitonin was detected in 13 patients (1.5%). In these 23 patients with a high calcitonin level, 11 (48%) were diagnosed with PTC, 10 (43%) with MTC, 1 (4.5%) with follicular patterned well-differentiated carcinoma (NOS), and 1 (4.5%) with nodular hyperplasia (Figure 3). Hypercalcitoninemia was detected in all 10 (100%) patients diagnosed with MTC. When the FNAB results were analyzed for the ten patients that were diagnosed with MTC histologically, the cytology of seven patients was compatible with suspicious for malignancy (Bethesda V), one was compatible with AUS/FLUS cytology (Bethesda III), and two with FN/FNS cytology (Bethesda IV). Of the cases with ND cytology, none of the patients were diagnosed with MTC.

**TABLE 1:**
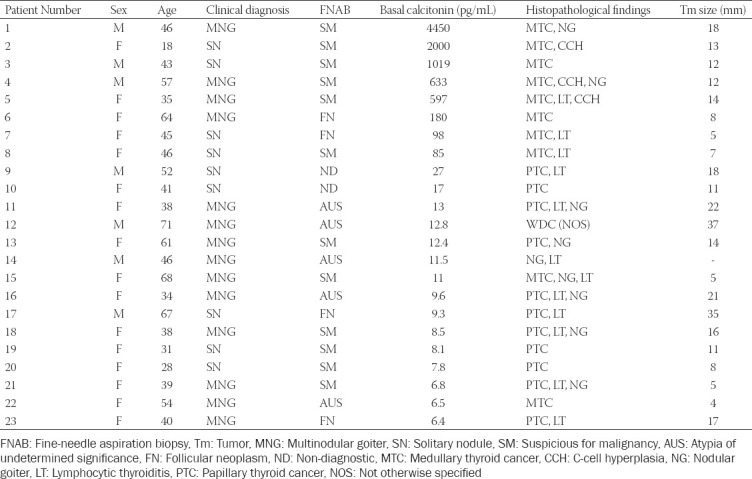
Clinical data, cytology results, preoperative calcitonin levels, and histological findings of patients with a high calcitonin level

## DISCUSSION

The aim of this study was to investigate the usefulness of calcitonin level measurement evaluated preoperatively in patients with nodular thyroid disease where the biopsy result is uncertain or suspicious. Of all the patients who underwent preoperative FNAB, 10 MTC patients who could not be diagnosed with cytology were identified. The pre-operative calcitonin level was above the assay specific cutoff value in all 10 MTC patients. Measurement of serum calcitonin is a sensitive and specific marker in the diagnosis and follow-up of MTC [13,15] and it is well known that the most important factor in MTC prognosis is early diagnosis of the disease [13,16]. Calcitonin measurement has been shown to be superior to FNAB in MTC detection in the previous studies [4,17,18] Nevertheless, routine calcitonin measurement in nodular thyroid disease is controversial. Although the detection of MTC through routine calcitonin screening has recently been shown to lead to early stage diagnosis, the ATA guideline did not make recommendations for routine calcitonin measurement in nodular thyroid patients, leaving the decision up to the clinicians [13]. However, many authorities recommend routine serum calcitonin measurement in nodular thyroid disease [4,7,9,10,17,19], but this recommendation is very controversial since it is considered that thyroid nodule prevalence is 33-68% [6,20-22]. In addition, the prevalence of MTC in nodular thyroid disease is unknown, and in thyroid cancers, its prevalence has been found to be 1-2% [1]. In the present study, the calcitonin level of all the histologically proven MTC patients was above the assay-specific cutoff value and false positive elevation of serum calcitonin was detected in 1.5% of patients, which may lead to unnecessary further investigation. Various physiological and pathological conditions other than medullary thyroid carcinoma (MTC) have been associated with increased calcitonin levels and one of these conditions is reactive or physiological C cell hyperplasia [22,23]. It has been hypothesized that the reason for the false positive elevation of serum calcitonin in these cases is reactive C cell hyperplasia due to lymphocytic thyroiditis, follicular cell-derived tumors or nodular hyperplasia [6,24-26]. Unfortunately, since calcitonin staining was not performed in these cases, C cell hyperplasia could not be confirmed, which can be considered a study limitation.

Although there are a few studies reporting that routine calcitonin measurement is cost-effective [27,28], routine calcitonin measurement does not seem rational for all nodular thyroid patients, considering the prevalence of medullary cancer. Perhaps the logical approach would be to measure calcitonin in selected cases [29]. The fact that 32-43% of European clinicians and only 2.5-4.9% of American clinicians use routine calcitonin measurement demonstrates that most clinicians use calcitonin measurement only in selected cases [30-32]. However, there are no data in the guidelines to state in which cases routine calcitonin measurement is more effective.

In the present study, none of the cases with multiple ND FNAB results were diagnosed with MTC histologically. The calcitonin measurement was of no value in these cases with multiple ND cytology. In the previous studies investigating the FNAB results of large medullary cancer case series, the ND/unsatisfactory rate has been found to be 0-4%, which is consistent with the results of the present study [33-35]. Calcitonin measurement helped diagnosis in 1 of 427 cases with AUS/FLUS cytology, and indicated MTC in 2 of 73 cases with FN/SFN cytology. An indeterminate cytology result was found at the rate of 0-30% in the previous studies investigating the FNAB results of large medullary cancer case series, which was similar to the current case series [4,18,33]. While routine calcitonin measurement seems to be reasonable in cases with FN/SFN, in cases with multiple AUS cytology, instead of routine calcitonin measurement, calcitonin can be screened particularly in patients with suspicious features of MTC on ultrasonography or with a family history of MTC [6].

The serum calcitonin level was above the assay-specific cutoff value in 12 out of 158 patients with cytology results indicating suspicion of malignancy. Of these patients, seven were diagnosed with MTC histologically. The measurement of calcitonin indicated the diagnosis of MTC and affected the extent of the surgery in these cases where total thyroidectomy was already indicated. In the previous studies, the capability of FNAB to detect MTC has ranged from 46% to 76% [33-35]. It is difficult to differentiate MTC from follicular cell-derived tumors cytologically. Therefore, the expected role of FNAB is not to reveal a definitive MTC diagnosis, but rather to indicate whether there is a suspicion of MTC [35]. As shown in this study, especially in cytologically suspicious cases, calcitonin measurement makes a great contribution to diagnosis and it is appropriate to use calcitonin measurement in these cases.

The main limitations of the current study were that stimulated calcitonin was not measured and the number of MTC cases was quite low. Therefore, studies with large multicenter case series are needed to evaluate the cost effectiveness of routine calcitonin measurement.

## CONCLUSION

Routine serum calcitonin measurement can be performed in selected cases rather than in all nodular thyroid patients. While routine calcitonin measurement is reasonable to use in patients with Bethesda IV and Bethesda V, routine calcitonin measurement in Bethesda I is not useful. In Bethesda III patients, patient-based decision can be made according to their calcitonin measurement.
